# Mechanical property enhancement in concrete composites with hybrid polypropylene fibre reinforcement

**DOI:** 10.1038/s41598-025-04219-6

**Published:** 2025-07-10

**Authors:** Chiang Ti Tan, Ming Kun Yew, Ming Chain Yew, Foo Wei Lee, Siong Kang Lim, Jing Han Beh, Jin Chai Lee, Jee Hock Lim

**Affiliations:** 1 Lee Kong Chian Faculty of Engineering and Science, Universiti Tunku Abdul Rahman, Bandar Sungai Long, Cheras, 43000 Kajang, Malaysia; 2 Department of Mechanical Engineering, Universiti Teknologi PETRONAS, Bandar Seri Iskandar, 32610 Perak Malaysia; 3 Department of Civil Engineering, UCSI University, Cheras, 56000 Kuala Lumpur, Malaysia

**Keywords:** Mechanical properties, Fibre-reinforced concrete, Hybrid polypropylene fibre, Composite materials, Recycled material, Civil engineering, Structural materials, Engineering, Materials science

## Abstract

Hybrid fibre-reinforced concrete is a specialised construction material known for its enhanced mechanical strength and durability. However, there is limited research available on hybridising three types of fibre in concrete. In this study, concrete was enhanced by incorporating three types of polypropylene (PP) fibre, namely Macro, Barchip and Monofilament PP fibre, with volume fractions of 0%, 0.1%, 0.2%, and 0.3%. Recycled granite powder was also used to partially replace fine aggregate to improve matrix density and promote sustainability. Results revealed that increasing fibre volume fractions significantly boosted mechanical properties. The Macro-Barchip-Monofilament (MBM) PP fibre mixture showed superior strength performance compared to the Barchip-Monofilament (BM) PP fibre mixture, with the MBM mix with fibre volume fractions of 0.3% achieving compressive, splitting tensile, and flexural strength improvements of 4.84%, 10.02%, and 15.83%, respectively. These enhancements were largely due to the combined effects of the hybrid PP fibres. While Barchip PP fibre effectively mitigated crack propagation, Macro PP fibre offered fine reinforcement to limit crack widths. Meanwhile, the dispersion of Monofilament PP fibre within the concrete matrix contributed to increased homogeneity. Nevertheless, the addition of hybrid PP fibres adversely affected workability, as evidenced by a 58.33% drop in slump values and an 84.62% rise in Vebe time. Furthermore, the ultrasonic pulse velocity (UPV) test highlighted advancements in concrete quality and scanning electron microscope (SEM) analysis demonstrated that fibre reinforcement enhanced the microstructure and bonding within the concrete. Post-cracking performance assessments confirmed the role of hybrid PP fibres in improving residual strength. Altogether, the findings indicate that hybrid PP fibre-reinforced concrete holds significant potential for creating durable and sustainable building materials.

## Introduction

Concrete materials are fundamental to the construction industry. However, traditional plain concrete is increasingly unable to satisfy the evolving demands of modern architecture due to its inherent drawbacks, such as low tensile strength and limited ductility. To overcome these challenges, considerable research has been conducted to enhance concrete properties. Among various approaches, incorporating fibres has emerged as an effective strategy to control crack propagation and enhance the properties of concrete. Nowadays, many studies have shown that there are several types of fibre that can be incorporated into concrete to enhance its properties, including steel, glass, basalt, polyvinyl alcohol, polypropylene, nylon, bamboo, kenaf, and other fibres^1–8^. Among these fibres, polypropylene (PP) fibres have emerged as one of the most widely utilised synthetic fibre types for reinforcing concrete, owing to their chemical inertness^9^, low density^10^, cost-effectiveness^11^, and resistance to corrosion^12^.

The presence of PP fibre in concrete is typically arranged in a randomly distributed and varied manner. By dispersing throughout the concrete matrix, PP fibres create a three-dimensional reinforcement network that helps to resist tensile stresses, preventing the formation and propagation of microcracks^13,14^. Beyond crack prevention, PP fibres also play a pivotal role in improving the post-cracking behaviour of concrete. Unlike conventional plain concrete, which exhibits brittle failure upon crack initiation, PP fibre-reinforced concrete maintains structural integrity even after cracks have formed^15,16^. When cracks occur, PP fibres have the ability to bridge across these cracks, which can distribute stress more effectively and enhance the concrete’s ductility. Moreover, PP fibres can also act as thermal insulators due to their polymeric nature. These fibres can create a discontinuous path for heat flow, disrupting direct conduction and improving the insulation of the concrete^17^.

In the concrete industry, there are several classifications of PP fibres based on their morphology and intended function, including Barchip PP, Macro PP, and Monofilament PP fibres, each contributing uniquely to the enhancement of concrete performance. Barchip PP fibre is a form of structured macro-synthetic fibre characterised by its embossed or deformed surface. Its strong bridging interaction with the cement matrix helps resist lateral tensile stresses generated during compressive loading^18^. Moreover, Macro PP fibre is typically longer and coarser than Monofilament PP fibre but thinner than Barchip PP fibre, designed to act as secondary reinforcement, which enhances energy absorption and toughness^19^. Monofilament PP fibre, being finer and more uniformly distributed, is known for its effectiveness in controlling early-age plastic shrinkage^20^ and microcrack formation^21^.

Although the incorporation of PP fibre improves various mechanical properties of concrete, it also has a significant impact on its workability. The presence of fibres increases the internal cohesion of the concrete mix, making it more resistant to flow and reducing its ease of placement and compaction. This reduction in workability is primarily due to the high surface area of the fibres, which increases water demand and hinders the movement of aggregate particles within the mix^22^. As the fibre volume fraction increases, the mix becomes stiffer and more challenging to handle. Therefore, the use of additional water or chemical admixtures is required to maintain adequate workability without compromising strength and durability.

Nowadays, there has been a growing interest in the concept of combining fibres, namely hybrid fibre-reinforced concrete (HFRC). HFRC is a specialised form of fibre-reinforced concrete that incorporates a combination of different types of discrete fibres within a cementitious matrix. Several studies have shown that the addition of hybrid fibre in concrete can improve the performance of concrete to a greater extent when compared with using single types of fibre^23–27^. By leveraging the unique properties of each fibre, a hybrid fibre system can effectively compensate for the limitations of single-fibre reinforcement. For example, a fibre with high tensile strength can efficiently bear the applied tensile load, while another fibre with high fracture toughness can effectively hinder crack propagation. When combined in concrete, they provide multidimensional reinforcement, enhancing both strength and durability. This interaction, commonly known as the synergistic mechanism, is gaining increasing recognition and adoption in the field of concrete.

For instance, the study by Zhou et al.^28^ found that when steel and polypropylene fibres were combined, the hybrid fibre system enhanced the ductility and toughness of concrete. Another study by Liang et al.^29^ observed there is a synergy effect between basalt fibres, which restrained microcrack initiation, and polypropylene fibres, which controlled macrocrack propagation, resulting in a more durable and ductile concrete matrix. Similarly, Ja’e et al.^30^ also revealed the synergistic effect of kenaf’s high stiffness and tensile properties, combined with polypropylene’s crack-bridging ability and shrinkage control​​. These studies have demonstrated the potential of hybrid fibre applications in concrete. However, current studies have mainly focused on hybrid fibre reinforcement using two types of fibres, while the combination of three different fibre types in concrete has not been extensively explored. There is a noticeable scarcity of data on hybrid fibres up to three distinct types.

Therefore, this study will focus on investigating the effects of hybridising three types of PP fibres, namely Barchip PP fibre, Macro PP fibre, and Monofilament PP fibre, on the mechanical performance and microstructural properties of high-strength concrete. Meanwhile, recycled granite powder (RGP), a byproduct of stone cutting, is used as a partial fine aggregate replacement to enhance bonding between fibre and cement matrix through its filler effect, owing to its finer and angular particles compared to sand^31^. This study aims to evaluate the influence of hybrid PP fibre reinforcement and RGP incorporation on enhancing the mechanical properties of high strength concrete.

## Materials and methodology

### Materials

#### Cement

In this study, Ordinary Portland Cement Type 1 (OPC) was utilised as the primary binder in the concrete mix. This versatile and multi-purpose cement is manufactured by YTL Cement Sdn. Bhd. under the brand name “Orang Kuat,” meets the MS EN 197-1:2014 CEM I 52.5 N standard requirements. To maintain its quality for future use, the cement was sieved using a 600 μm sieve and stored in an airtight container to prevent moisture absorption and contamination.

#### Aggregate

**Fig. 1:**
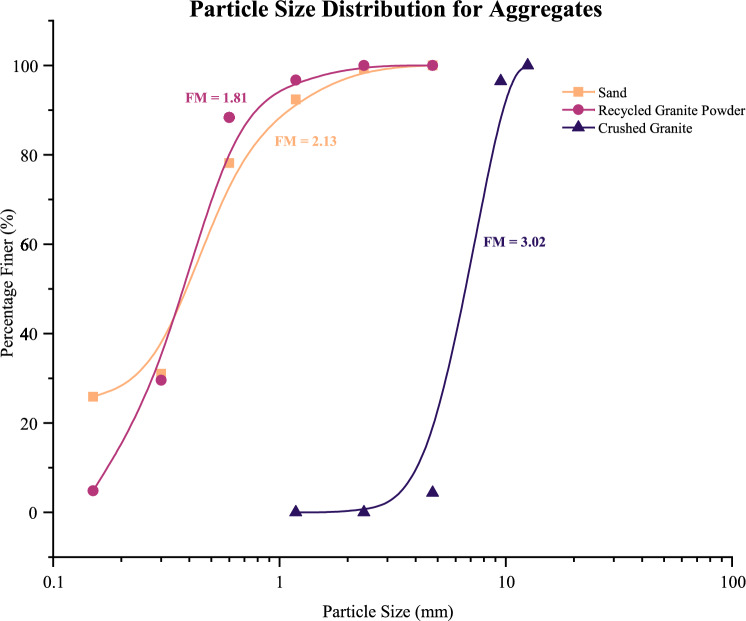
Particle size distribution curve for sand, recycled granite powder and crushed granite.

**Fig. 2:**
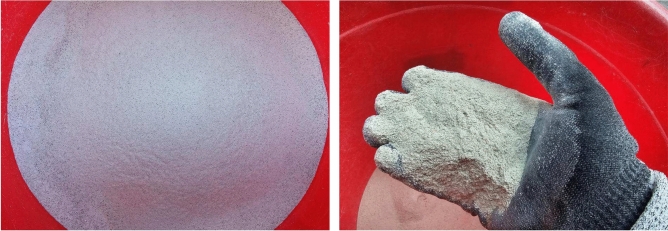
Granite powder used in this study.

This study involved the utilisation of three (3) distinct aggregates, namely local mining sand, recycled granite powder, and crushed granite. The particle size distribution curve for the aggregates is shown in Fig. 1. The mining sand was sieved to a maximum particle size of 4.75 mm and air-dried before use, with a specific gravity of 2.52 and a fineness modulus of 2.13. Additionally, recycled granite powder, a byproduct of granite crushing, was incorporated as an alternative fine aggregate, as shown in Fig. 2. It was sieved through a 600 μm sieve and stored in a container to maintain its quality. For coarse aggregate, the granite was processed by passing it through a 10 mm sieve to ensure particle sizes met the requirements for fibre-reinforced concrete, while particles smaller than 4.75 mm were removed. To eliminate moisture variation, the aggregate was dried in an open environment for at least 24 h. The crushed granite had a specific gravity of 2.75 and a fineness modulus of 3.02, making it suitable for concrete applications.

#### Water and superplasticizer

Potable water was used for both the mixing and curing processes to ensure consistency and quality in concrete production. To enhance the workability of the concrete during mixing, MasterGlenium ACE 8538 superplasticiser was incorporated. In accordance with standard guidelines, the superplasticiser was applied at a dosage of 1.5% by weight of the cementitious binder, ensuring optimal dispersion and effective hydration of the cement particles.

#### Polypropylene fibre

**Fig. 3:**
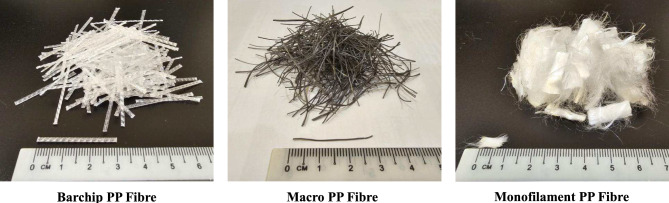
Polypropylene fibres used in this study.

This study incorporates three distinct types of polypropylene fibres: Barchip, Macro, and Monofilament polypropylene fibres, as shown in Fig. 3. The physical properties of these fibres are detailed in Table 1. The Monofilament polypropylene fibre, measuring 6 mm in length, will be utilised. Meanwhile, the Macro and Barchip polypropylene fibres will be cut to a length of 30 mm before being integrated into the concrete mixture.

**Table 1:** Physical properties of the polypropylene fibre.

Properties	Barchip PP fibre	Macro PP fibre	Monofilament PP fibre
Length (mm)	30	30	6
Diameter (mm)	1	0.3	0.03
Density (kg/m^3^)	910	910	910
Specific gravity	0.91	0.91	0.91
Tensile strength (MPa)	550	460	450
Elastic modulus (GPa)	9	6	–

### Mix design

The study utilised a cement: fine aggregate (FA): coarse aggregate (CA) ratio of 1:2.3:2.25 and a water-cement ratio of 0.35. To enhance the concrete properties, recycled granite powder (RGP) is used as a partial replacement for fine aggregate, with previous findings showing a 5–10% improvement in strength. Therefore, a 5% replacement level is selected for all mixes to balance performance and workability^32^. Additionally, polypropylene (PP) fibres will be incorporated in two different combinations with volume fractions of 0%, 0.1%, 0.2%, and 0.3%. The mix proportions for each material are detailed in Table 2.

**Table 2:** The mix proportion of each mix code.

Mix code	Material (kg/m^3^)								
	Cement	FA	CA	RGP	Water	SP	Macro PP fibre (%)	Barchip PP fibre (%)	Monofilament PP fibre (%)
CT	414	903	935	46	147	6.9	–	–	–
MBM-1	414	903	935	46	147	6.9	0.025	0.025	0.05
MBM-2	414	903	935	46	147	6.9	0.05	0.05	0.1
MBM-3	414	903	935	46	147	6.9	0.075	0.075	0.15
BM-1	414	903	935	46	147	6.9	–	0.05	0.05
BM-2	414	903	935	46	147	6.9	–	0.1	0.1
BM-3	414	903	935	46	147	6.9	–	0.15	0.15

### Testing method

In this study, various tests were conducted to evaluate the properties of the concrete mixtures. The workability of the mixtures was assessed using both slump and Vebe tests to determine the ease of placement and compaction. Additionally, the fresh density of the concrete was measured to ensure uniformity and consistency in the mix. The mechanical performance of the concrete was examined through compressive, splitting tensile, and flexural strength tests to monitor strength development over time. The testing machine utilised for mechanical performance is ADS 300/EL, which was produced by Unit Test Scientific Sdn. Bhd. and possessed a load capacity of 3000 kN. The residual compressive strength and splitting tensile strength tests were performed by subjecting the specimens to repeated loading cycles with a controlled failure rate of 10% to evaluate post-crack performance.

**Fig. 4:**
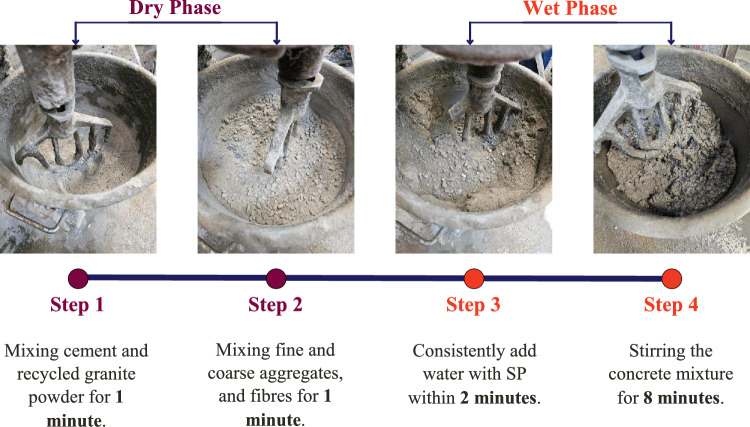
Flow of 4-step mixing method.

In order to further analyse the internal structure of the concrete, an ultrasonic pulse velocity (UPV) test was conducted to assess material integrity and uniformity. Moreover, a scanning electron microscope (SEM) test was also used to study the microstructural characteristics, focusing on the fibre-matrix interaction and crack-bridging mechanisms. The SEM test will be conducted through the Hitachi S-3400N VP-SEM, which is manufactured by Hitachi High-Tech Corporation. The standard criteria for performing HFRC tests are outlined in Table 3. The sample size and testing ages for each test are shown in Table 4. This study will utilise the 4-step mixing approach for concrete mixing, as illustrated in Fig. 4.

**Table 3:** The standard code for each test.

Test	Standard
Slump test	BS EN 12350-2: 2019^33^
Vebe test	BS EN 12350-3: 2019^34^
Fresh density test	BS EN 12350-6: 2019^35^
Compressive strength test	BS EN 12390-3: 2019^36^
Splitting tensile strength test	BS EN 12390-6: 2023^37^
Flexural strength test	BS EN 12390-5: 2019^38^
Ultrasonic pulse velocity (UPV) test	BS EN 12504-4: 2021^39^

**Table 4:** The sample size and testing ages for each test.

Test	Specimen type	Dimension (mm)				Testing age (days)			
		Length	Width	Height	Diameter	7	28	56	
Compressive strength	Cubic	100	100	100	–	3	3	3	No of sample
Splitting tensile strength	Cylindrical	200	–	–	100	3	3	–	
Flexural strength	Prismatic	500	100	100	–	3	3	–	
Ultrasonic pulse velocity test	Cubic	100	100	100	–	3	3	3

## Results and discussion

### Density

**Fig. 5:**
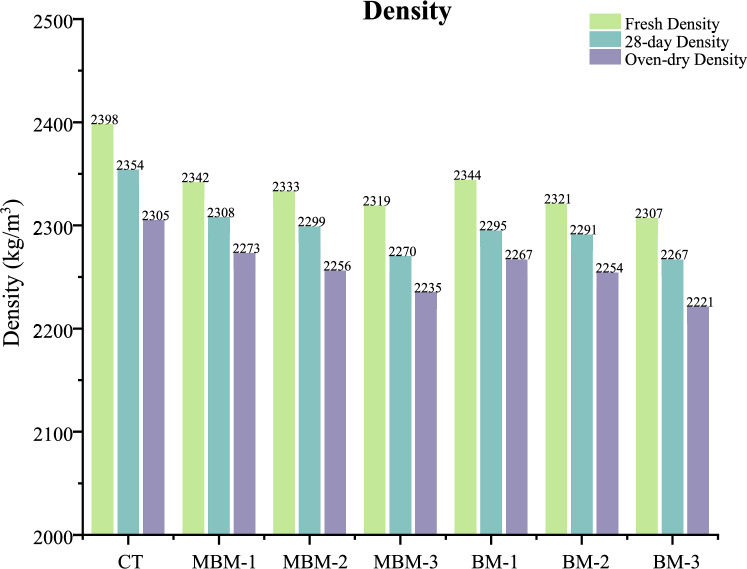
Density of each mix.

The variation in concrete density across different mixes for three density states: fresh density, 28-day density, and oven-dry density, are shown in Fig. 5. A clear decreasing trend is observed as the fibre volume fraction increases, indicating that polypropylene fibre incorporation leads to a reduction in density. The CT mix exhibits the highest density in all three states, with a fresh density of 2398 kg/m^3^, a 28-day density of 2354 kg/m^3^, and an oven-dry density of 2305 kg/m^3^. As the fibre volume fraction increases, the density values progressively decrease. Among all the mixes, BM-3 records the lowest densities, with a fresh density of 2307 kg/m^3^, a 28-day density of 2267 kg/m^3^, and an oven-dry density of 2221 kg/m^3^. The reduction in density is primarily attributed to the lightweight nature of polypropylene fibres, which replace denser concrete materials like aggregate and cement^40^. This finding aligns with the study by Yew et al.^41^, which concluded that the lower specific gravity of polypropylene fibre will cause a reduction in density.

The slight fluctuations in density across MBM and BM mixes indicate that different polypropylene fibre types influence the density reduction in a similar manner, as both have an equivalent density of 0.91 kg/m3. Despite these variations, the overall trend shows that increasing fibre content leads to lower concrete density, reinforcing the lightweight characteristics of fibre-modified concrete.

### Workability

**Fig. 6:**
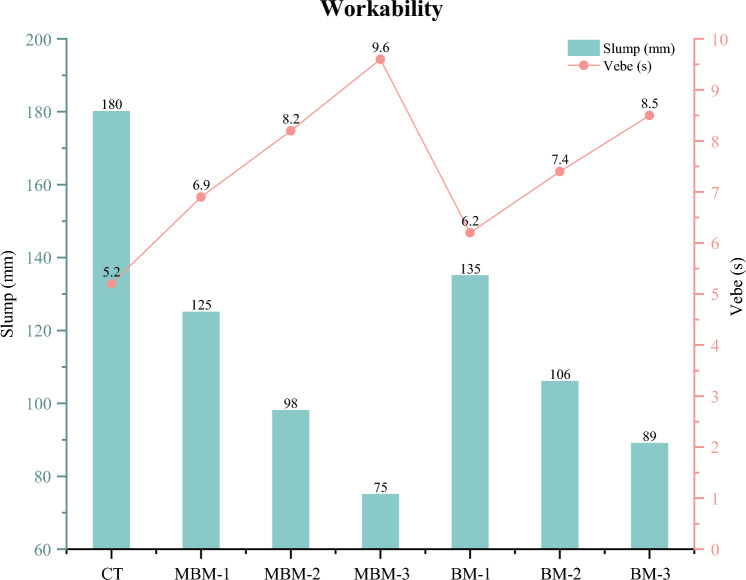
Workability of each mix.

**Fig. 7:**
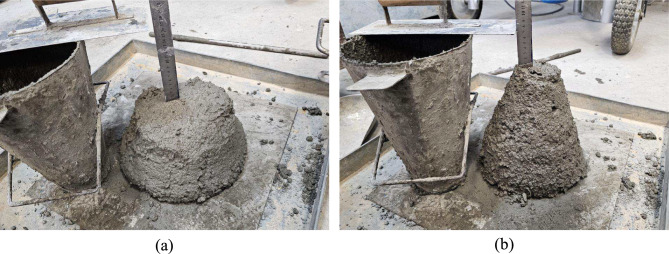
Effect of fibre addition on concrete workability: (**a**) without fibre, (**b**) with 0.3% fibre.

The effect of hybrid polypropylene fibre incorporation on the workability of fresh concrete, as measured by the slump test and vebe test, is shown in Fig. 6. It is evident that as fibre content increases, the slump value decreases while the vebe time increases, indicating a reduction in workability, as shown in Fig. 7. The CT mix exhibited the highest slump at 180 mm and the shortest vebe time of 5.2 s. However, as fibre content increased, a significant drop in slump was observed, with MBM-3 recording the lowest slump at 75 mm, representing a 58.33% reduction. Simultaneously, the vebe time increased from 5.2 to 9.6 s in MBM-3, marking an 84.62% rise. This trend highlights the restrictive effect of fibres on concrete movement, as they create an interlocking network that impedes the free flow of the mix^42,43^.

The reduction in workability with fibre incorporation is primarily attributed to the increased surface area within the concrete matrix. As fibres are added, a larger quantity of cement paste is required to coat them, reducing the available paste for inter-particle lubrication^22,44^. This results in higher viscosity and lower fluidity, making the mix more resistant to flow. Furthermore, comparing different fibre types, it is observed that MBM mixes experienced a greater drop in slump values and a higher rise in vebe times compared to BM mixes. This suggests that Macro PP fibre has a more pronounced impact on increasing concrete viscosity due to its greater tendency to clump together, further restricting flowability. A comparable conclusion was also made by Bentegri et al.^45^, whose study stated that Macro PP fibre exacerbates this negative effect on workability by promoting fibre entanglement, leading to increased internal friction within the mix.

### Ultrasonic pulse velocity

**Fig. 8:**
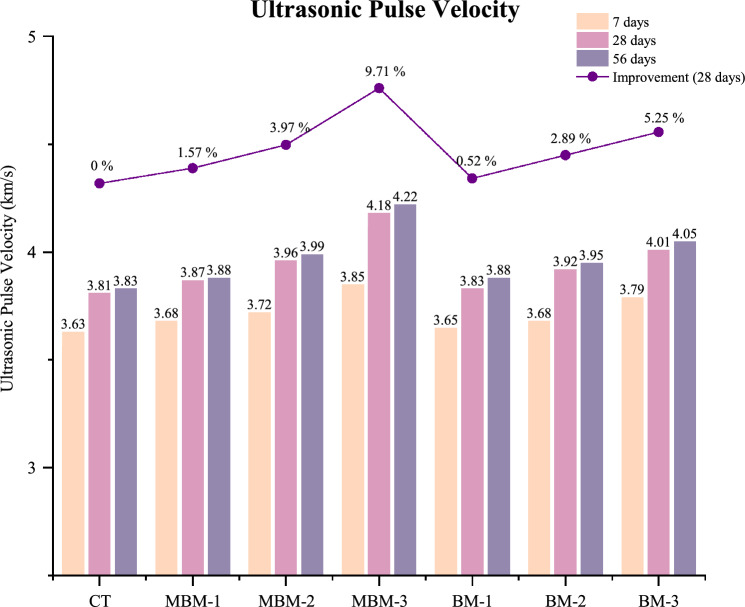
Ultrasonic pulse velocity of each mix.

The UPV test was employed to assess concrete quality and identify internal cracks by measuring the velocity of ultrasonic waves passing through the material. In general, a higher UPV value indicates better concrete quality, characterised by fewer voids, increased density, improved integrity, and greater homogeneity. As illustrated in Fig. 8, the UPV values range between 3.81 and 4.18 km/s. Thus, all the concrete mixes can be classified as having “good” to “excellent” quality based on BS EN 12504-4. Among all specimens, the CT mix recorded the lowest UPV values across all curing ages, with a UPV of 3.81 km/s at 28 days. This suggests that concrete without fibre reinforcement is more prone to internal voids and microcracking, leading to reduced wave transmission efficiency^46^. In contrast, MBM-3 exhibited the highest UPV at 28 days (4.18 km/s), marking a 9.71% improvement compared to the CT Mix. This enhancement is attributed to the filler effect of RGP, which helps fill micro-voids in the concrete, improving the bond between the fibres and the cement matrix. Meanwhile, the incorporation of hybrid PP fibre, which effectively bridges cracks, allows for more efficient transmission of sound waves. Similar findings were reported by Savio et al.^47^, reinforcing the notion that incorporating fibres resulted in higher UPV.

Additionally, all specimens displayed a slight increase in UPV values from 28 to 56 days, aligning with the ongoing hydration process. However, the improvement rate was relatively minor, as most hydration reactions slowed down significantly after 28 days. A similar trend was reported by Shahjalal et al.^48^. This behaviour is attributed to the hydration process, which is most active during the early curing period, leading to rapid microstructural development. However, as the concrete continues to age, the hydration reaction slows down, resulting in a lower rate of pulse velocity increase. Notably, at an equivalent fibre volume fraction, MBM mixtures achieved higher UPV values than BM mixtures, which suggests that the presence of both Macro and Monofilament PP fibre provides better crack resistance than Barchip PP fibre. The higher proportion of finer fibres in MBM likely contributed to improved crack bridging and better distribution within the concrete, reducing the formation and propagation of microcracks.

### Compressive strength

**Fig. 9:**
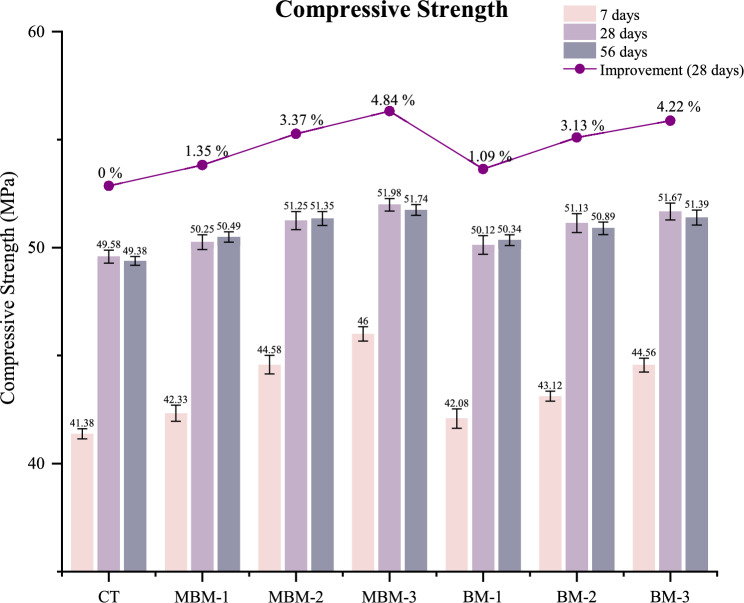
Compressive strength of each mix. Error bars represent standard deviation.

The compressive strength test is a fundamental method for assessing the strength of hardened concrete. As illustrated in Fig. 9, The CT mix achieved a 28-day compressive strength of 49.58 MPa, while all hybrid PP fibre-reinforced mixes exhibited higher strengths, ranging between 50.25 and 51.98 MPa. The most significant improvement in 28-day strength is observed in MBM-3, which shows a 4.84% increase compared to CT. The improvement in compressive strength can be attributed to the crack-bridging ability of fibres, as noted by Khaloo et al.^49^. The incorporation of polypropylene fibres enhances stress distribution within the concrete matrix, restricting crack propagation and improving performance under applied loads.

Among the reinforced mixes, the MBM combinations consistently exhibited higher compressive strength improvements compared to BM combinations. This trend highlights the effectiveness of Macro PP fibre and Monofilament PP fibre, as fibres with smaller diameters demonstrated greater efficiency in bridging cracks and enhancing compressive strength compared to larger fibres^50^. However, the overall improvement in strength remains moderate, ranging from 1.09% to 4.84%, indicating that excessive fibre addition does not significantly enhance compressive strength. This finding aligns with previous studies by Khan et al.^51^ and Zhong and Zhang^52^, which also reported that an excessive fibre dosage does not necessarily yield substantial strength gains. Additionally, the 56-day compressive strengths remain relatively similar to their 28-day counterparts, with differences within 0.3 MPa. This suggests that the presence of PP fibre helps sustain long-term strength while reducing deterioration over time^53^.

**Fig. 10:**
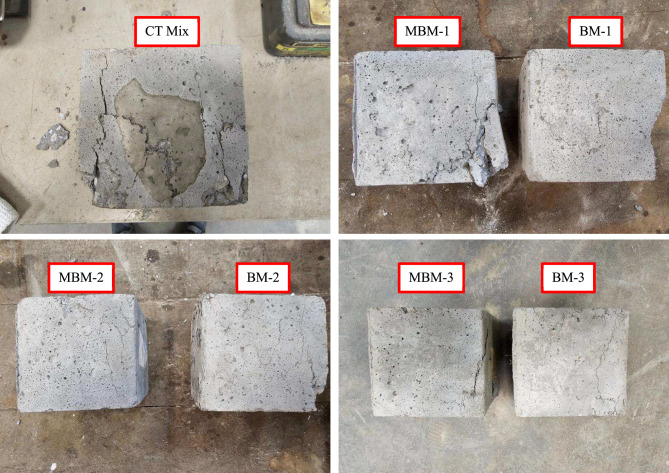
Failure mode of the concrete cube after loading.

The contrasting failure behaviours of plain and fibre-reinforced concrete become evident during compression testing. Plain concrete exhibits a brittle failure mode, where cracking leads to instantaneous disintegration due to its limited tensile load-bearing capacity. In contrast, HFRC demonstrates greater resilience and ductility^54^. Even after the onset of cracking, the fibre network within HFRC helps maintain structural integrity. These fibres act as a binding mechanism, enhancing the concrete’s ability to withstand compressive stresses while reducing the likelihood of sudden failure. For instance, the Monofilament PP fibre can bridge across the micro cracks to prevent the micro crack from becoming a macro crack. Even though macro-cracks may still develop, both Macro and Barchip PP fibres can effectively bridge these cracks. This synergistic action transforms the brittle failure mode of concrete into a more ductile response. This enhanced ductility is reflected in the failure pattern shown in Fig. 10, highlighting the beneficial role of hybrid fibres in improving energy absorption and overall mechanical performance.

### Residual compressive strength

**Fig. 11:**
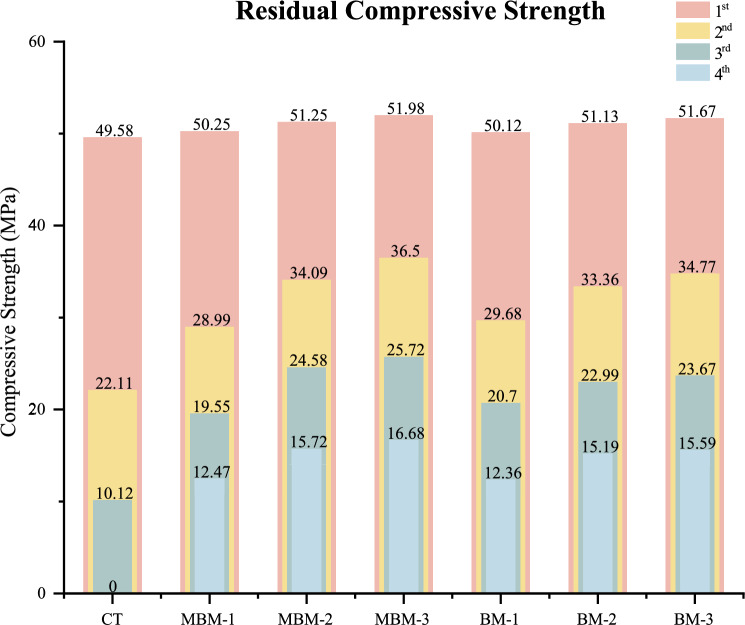
Residual compressive strength of each mix.

The residual compressive strength test evaluates the impact of hybrid fibre incorporation on concrete’s ability to retain strength post-failure. This study measures residual strength across four stages, with the initial stage determining maximum compressive strength and subsequent stages assessing the concrete’s load-bearing capacity after cracking. As shown in Fig. 11, the CT mix exhibits a sharp decline in residual compressive strength, reducing to 22.11 MPa, 10.12 MPa, and ultimately 0 MPa in the 2nd, 3rd, and 4th stages, respectively. In contrast, the MBM-3 mix demonstrates significantly higher retention, with residual strengths of 36.5 MPa, 25.72 MPa, and 16.68 MPa, highlighting the positive effect of incorporating fibre. Notably, increasing the fibre volume fraction from 0.1% to 0.3% in the concrete leads to substantial improvements, with the 2nd, 3rd, and 4th stage residual strengths rising from 28.99 to 36.5 MPa, 19.55 to 25.72 MPa, and 12.47 to 16.68 MPa for MBM mix, respectively.

The integration of hybrid PP fibres can contribute to residual compressive strength enhancement by bridging cracks, restricting their propagation and improving the concrete’s post-failure load-bearing capacity. Additionally, the synergistic effect of different fibre types distributes stresses more effectively, increasing ductility and energy absorption, which helps the concrete sustain multiple stages of damage before complete failure. These findings indicate that a higher fibre volume fraction enhances the concrete’s ability to withstand progressive damage, aligning with the results of Loh et al.^55^ and Yong et al.^17^.

### Splitting tensile strength

**Fig. 12:**
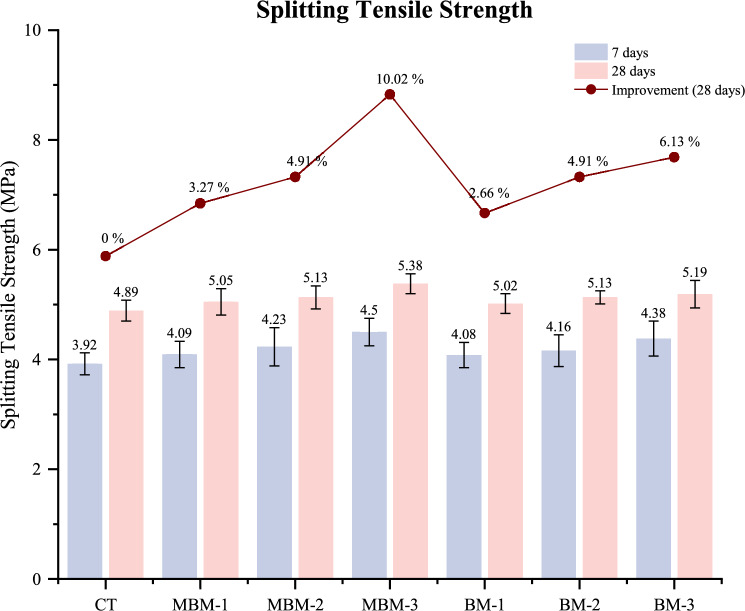
Splitting tensile strength of each mix.

Hybrid PP fibre is known to enhance the tensile properties of concrete. Thus, conducting the splitting tensile strength test is essential to determining the effectiveness of hybrid PP fibre in reinforcing concrete. As illustrated in Fig. 12, the 28-day splitting tensile strength of all concrete mixes ranges from 4.89 to 5.38 MPa, with the CT mix displaying the lowest value at 4.89 MPa. Among the tested mixes, MBM-3 achieves the highest splitting tensile strength at 28 days, reaching 5.38 MPa, representing a 10.02% improvement over the CT mix. An increase in fibre volume fraction from 0.1 to 0.3% leads to a noticeable improvement in splitting tensile strength in both MBM and BM combinations. These improvements are attributed to PP fibre’s ability to bridge cracks within the concrete matrix. As cracks initiate and propagate, the fibres transfer stress from the matrix to themselves, delaying fracture and increasing the concrete’s tensile strain capacity^56^.

Specifically, the MBM combination consistently outperforms the BM combination, showing higher tensile strength than their BM counterparts. The higher tensile strength of MBM-3 (5.38 MPa) compared to BM-3% (5.19 MPa) suggests that the synergistic effect of hybrid fibres plays a crucial role. For instance, Barchip PP fibre can enhance crack bridging^57^, while Macro PP fibre functions as fine reinforcement to prevent cracks and control their width. Additionally, Monofilament PP fibre improves matrix homogeneity, further contributing to enhanced tensile resistance.

**Fig. 13:**
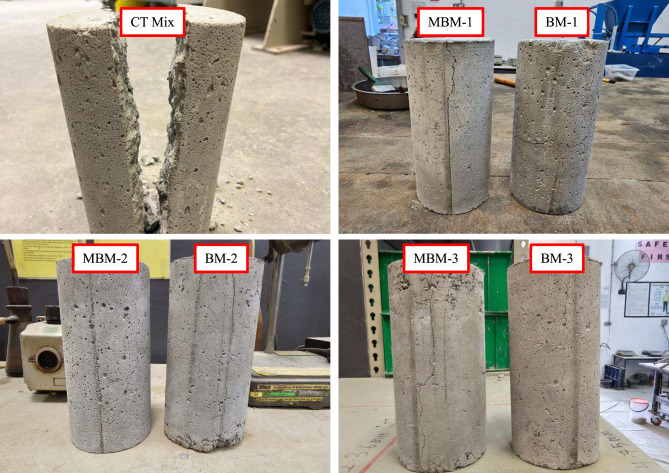
Failure mode of the concrete cylinder after loading.

During the splitting tensile test, the CT mix was fractured into two separate pieces, whereas HFRC specimens remained intact, with the fractured sections still connected. Figure 13 demonstrates that fibre volume fractions of 0.1% and 0.2% exhibit moderate fracture resistance, keeping the specimens linked after failure, while 0.3% fibre content significantly enhances fracture resistance by reducing fracture line width. This result highlights the effective bridging effect of hybrid PP fibre, which binds concrete layers together, improves internal confinement, and resists lateral expansion under stress. Consequently, incorporating hybrid PP fibre strengthens the concrete’s ability to endure tensile stresses, reinforcing its resistance to cracking.

### Residual splitting tensile strength

**Fig. 14:**
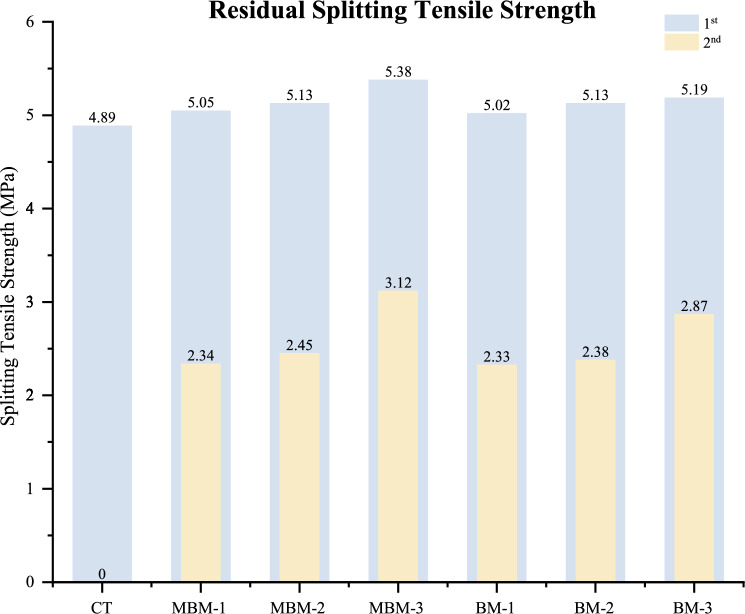
Residual splitting tensile strength of each mix.

The residual splitting tensile strength test for HFRC assesses the effect of hybrid fibre inclusion on concrete’s ability to withstand splitting stresses after initial damage. This test is conducted in two stages, with the first stage determining the maximum splitting tensile strength—the point where concrete starts to fail under perpendicular tensile stress. The second stage, known as the residual splitting tensile strength stage, evaluates the concrete’s capacity to sustain tensile loading beyond the initial fracture until complete failure. As shown in Fig. 14, the CT mix fails completely after the first stage, exhibiting a residual splitting tensile strength of 0 MPa. In contrast, the MBM-3 mix retains approximately 58% of its initial splitting tensile strength, demonstrating the highest residual strength among all mixtures. Furthermore, an increase in fibre volume fraction from 0.1 to 0.3% in the concrete leads to a rise in residual splitting tensile strength from 2.34 to 3.12 MPa for the MBM mix, confirming that a higher fibre volume fraction enhances HFRC’s ability to resist tensile failure.

The residual splitting tensile strength of HFRC can be attributed to the bridging effect of hybrid PP fibres, particularly Barchip PP fibre, which prevents crack widening and enables the concrete to sustain loads even after reaching its failure point^55^. Unlike plain concrete, which fractures completely after the second-stage loading, HFRC maintains structural integrity due to the ability of fibres to restrain crack propagation. This bridging effect helps the concrete retain post-failure strength, while Macro PP fibre further contributes by inhibiting crack formation. The synergistic effect of these fibres plays a crucial role in enhancing the post-failure performance of HFRC.

### Flexural strength

**Fig. 15:**
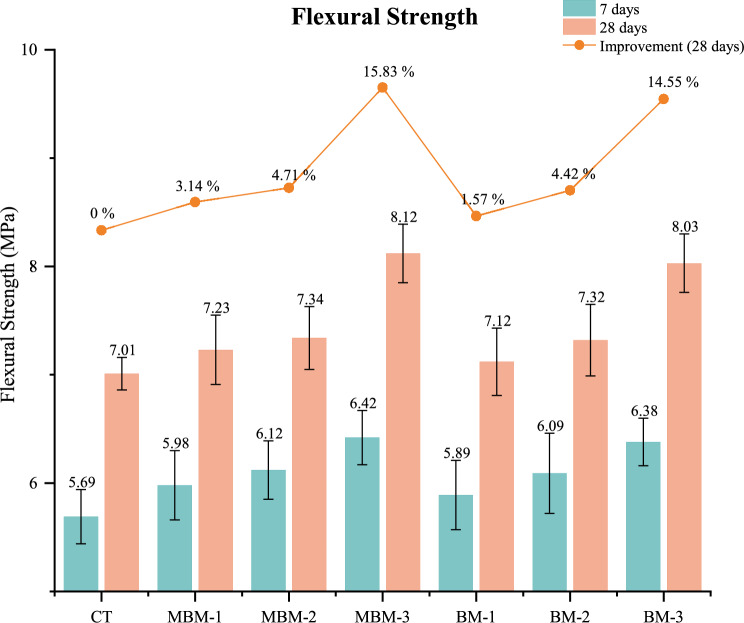
Flexural strength of each mix. Error bars represent standard deviation.

The flexural strength of various concrete mixes at 7 and 28 days, are shown in Fig. 15. At 28 days, the flexural strength of the CT mix is 7.01 MPa, whereas the MBM and BM combinations show incremental improvements as the fibre volume fraction increases from 0.1% to 0.3%. The MBM-3 mix exhibits the highest flexural strength at 8.12 MPa, representing a 15.83% improvement over the CT mix. Similarly, the BM-3 mix achieves 8.03 MPa, marking a 14.55% enhancement. This enhancement in flexural strength is primarily attributed to the fibre reinforcement mechanism, where fibres bridge across cracks and delay their propagation. As these fibres stretch, they introduce an additional energy absorption mechanism within the material, improving its resistance to flexural failure^58^. The observed trend aligns with findings from previous studies by Ahmad et al.^59^, reinforcing the role of fibre reinforcement in enhancing flexural properties.

**Fig. 16:**
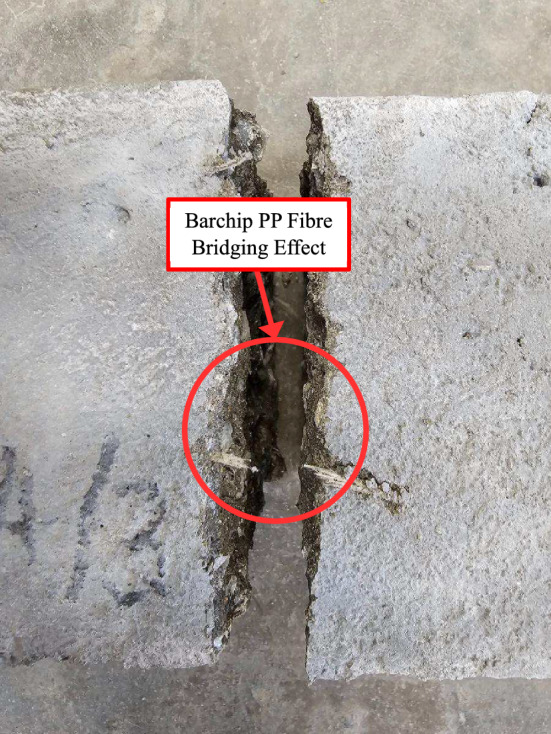
Failure mode of prism with fibre bridging effect.

Furthermore, the result reveals that the MBM series demonstrates higher overall flexural strength compared to the BM series, indicating that the synergistic effect of combining Macro and Monofilament PP fibre with Barchip PP fibre results in better performance. The MBM-3 mix shows the most significant enhancement, likely due to the effective interaction of different fibre types in preventing crack formation and controlling crack width. Specifically, the incorporation of Barchip PP fibre can hinder crack propagation by absorbing and distributing the tensile stresses induced by bending^60^, as depicted in Fig. 16. This multi-fibre reinforcement strategy strengthens the concrete’s ability to resist fracture opening and expansion, ultimately leading to improved flexural strength.

### Scanning electron microscope (SEM)

**Fig. 17:**
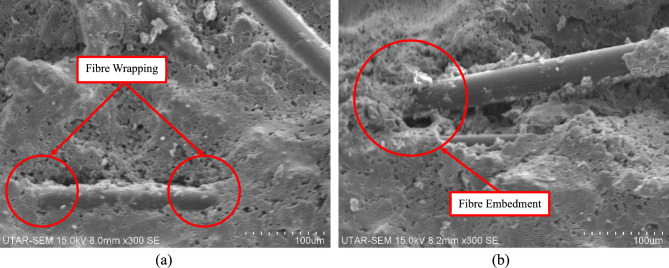
(**a**) Fibre wrapping effect. (**b**) Fibre embedment effect.

In this study, the Scanning Electron Microscope (SEM) was employed as a powerful imaging technique to examine the microstructure of polypropylene fibre-reinforced high-strength concrete at high magnification, focusing on the fibre-matrix bonding quality. As illustrated in Fig. 17, a substantial amount of hydration products were found to adhere to the surface of polypropylene fibres, indicating a strong interfacial bond between the fibres and concrete particles. This bond plays a critical role in enhancing tensile strength and crack resistance, as supported by the findings of Liu et al.^61^ and Tan et al.^62^, which also demonstrate a tight connection between PPF and the cement matrix. The presence of hydration products suggests that the fibres actively participate in the hydration process, improving mechanical properties and durability. Additionally, Fig. 17 highlights the fibre-bridging effect, where the embedment of polypropylene fibres strengthens the concrete by securely anchoring the fibres, enabling the effective transfer and distribution of internal stresses along their length. This bridging mechanism helps resist external forces, promotes uniform stress distribution, and mitigates localised failure, ultimately enhancing the overall load-bearing capacity and structural integrity of high-strength concrete.

## Conclusion

Based on the analysis results and the subsequent discussion, the following conclusion can be drawn:

1. Incorporating hybrid PP fibres reduced concrete density, with oven-dry density ranging from 2221 to 2273 kg/m^3^ compared to the CT mix at 2305 kg/m. Despite this reduction, the hybrid PP fibres enhanced crack resistance and strength, ensuring that the concrete maintained its strength while decreasing its weight.
2. The inclusion of hybrid PP fibres significantly reduces concrete workability, as seen in the 58.33% slump reduction and 84.62% increase in vebe time for the MBM-3 mix due to increased surface area and interlocking effects. Macro PP fibre further intensifies this impact by promoting fibre entanglement, necessitating superplasticiser use to maintain desired workability levels.
3. The incorporation of hybrid PP fibres enhanced UPV values, with HFRC showing an increase ranging from 0.52 to 9.71% over the CT mix. This enhancement is due to the strong bonding between the fibre and cement and the synergistic effect of PP fibres, which improve crack resistance and promote a denser structure.
4. The addition of hybrid PP fibre led to little enhancements in compressive strength, increasing by 1.09–4.84% at 28 days. This is attributed to the bridging and synergistic effect of hybrid PP fibres, which can significantly mitigate cracking and improve the failure mechanism of the concrete from brittle to ductility.
5. Incorporating PP fibres led to a significant enhancement in 28-day splitting tensile strength, ranging from 2.66% to 10.02%. Likewise, the 28-day flexural strength increased by 1.57% to 15.83% compared to the control sample. This enhancement is due to the hybrid PP fibre’s ability to interconnect cracks, effectively dispersing stress and increasing resistance to fracture.
6. The MBM mix shows better performance compared to the BM mix. This could be attributed to the larger diameter of Barchip PP fibre, which makes uniform dispersion within the mix more challenging. In contrast, the incorporation of Macro PP fibre with a smaller diameter helps mitigate this issue, ensuring better fibre distribution.
7. The residual strength tests revealed that hybrid PP fibres significantly improve concrete’s post-failure performance. In the residual compressive strength test, increasing the fibre volume fraction from 0.1% to 0.3% enhanced the 2^nd^, 3^rd^, and 4th stage strengths by approximately 25.9%, 31.6%, and 33.8%, respectively, while the residual splitting tensile strength increased by 33.3% for MBM mix.
8. The scanning electron microscope (SEM) analysis revealed a strong fibre-matrix bond in polypropylene fibre-reinforced high-strength concrete, evidenced by hydration products adhering to the fibre surface, which enhances tensile strength and crack resistance.

## Data Availability

The authors confirm that the data supporting the findings of this study are available within the manuscript.
